# Permanent atrial fibrillation ablation surgery in patients with advanced age

**Published:** 2005-10-01

**Authors:** Stephan Geidel, Michael Lass, Ostermeyer Jörg

**Affiliations:** Department of Cardiac Surgery, AK St. Georg, Hamburg, Germany

**Keywords:** Atrial fibrillation, atrial fibrillation surgery, radiofrequency ablation, arrhythmia surgery, bipolar radiofrequency ablation

## Abstract

**Background:**

Even if permanent atrial fibrillation (pAF) is a frequent concomitant problem in patients undergoing open heart surgery and particularly in those with advanced age, data of pAF ablation surgery in older aged patients are scarce. This study was performed to assess early and late results of combined open heart surgery and pAF ablation procedures in patients with advanced aged, compared to young patients.

**Material and Methods:**

A selective group of 126 patients (Group A: age =70 [76.4±4.8] years, n=70; Group B: age <70 [62.0±6.2] years: n=56) with pAF (=6 months) underwent either monopolar (Group A, B: n=51 vs. n=44) or bipolar (Group A, B: n=19 vs. n=12) radiofrequency (RF) ablation procedures concomitant to open heart surgery. Regular follow-up was performed 3 to 36 months after surgery to assess survival, New York Heart Association (NYHA) class and conversion rate to stable sinus rhythm (SR).

**Results:**

Early mortality (<30 days) was 2.9% in Group A (Group B: 0%), cumulative survival at long-term follow up was 0.78 vs. 0.98 (p=0.03) and NYHA-class improved significantly in both groups, particularly in cases with stable SR. At 12-months follow-up 73% of Group A patients were in stable SR (Group B 78%).

**Conclusion:**

Concomitant mono- and bipolar RF ablation surgery represents a safe option to cure pAF during open heart surgery with a very low risk, even in patients with advanced age.

## Introduction

Permanent atrial fibrillation (pAF) is a serious concomitant problem in many patients undergoing open heart surgery, particularly in those with advanced age: a prospective analysis among 4590 patients, which were scheduled for open heart surgery in our institution between February 2001 and December 2004, revealed a prevalence of pAF (persisting for at least 6 months) of 4.6% in older aged patients (70-99 years), compared to only 2.9% in young patients (p<0.001; Table 1); the prevalence was particularly high in elderly women (7.2% [58 of 809], compared to 3.1% [43 of 1381] in older aged men; p<0.001).

As pAF significantly deteriorates the prognosis particularly at advanced age [1], reliable ablation concepts are required to eradicate pAF during open heart surgery in these patients. This study was performed to assess early and late results (survival, improvement of New York Heart Association [NYHA] class and SR conversion rate) of combined open heart surgery and pAF ablation procedures using monopolar and bipolar radiofrequency (RF) energy in patients with advanced aged, compared to young patients.

## Patients and methods

### Patients

The population existed of a selective group of 126 patients (Group A: age =70 [76.4±4.8] years, n=70; Group B: age <70 [62.0±6.2] years: n=56) with pAF (=6months) which were scheduled for open heart surgery in our department between February 2001 and December 2004. Exclusion criteria for ablation surgery were: emergency operation, Euro-score >12 (*Euro-score: established score to evaluate the predicted risk of  cardiac operations [low risk: 1-2 points; moderate risk: 3-5 points; high risk: =6 points]*), severe reduced left ventricular function (LVEF =25%), acute endocarditis or myocardial infarction (=7days), considerable cachexia (Body Mass Index =18), intracardiac thrombosis with one or more large (>20mm) floating thrombus, left atrial (LA) enlargement with an LA-diameter of =72mm in the  transthoracic echocardiography (parasternal M-mode acquisition) or any other form of AF (intermittent or permanent lasting less than 6 months). All relevant data of patient characteristics are given in Table 2. There was no significant difference of NYHA class, left ventricular (LV) ejection fraction (EF) or LA-size between Group A and B, but Group A patients had a significantly higher Euro-score and a shorter duration of pAF duration. In contrast to Group B, most Group A patients were female. All patients were managed medically before and after surgery with standard heart failure medications, 6 of Group A patients had beta-blockers (Group B: n=4), 6 patients were on amiodarone (Group B: n=6).

### Mono- and bipolar RF ablation surgery, perioperative management and follow-up

The *monopolar endocardial* procedure has been described before [1]. From February 2001 to February 2003 all patients scheduled for combined heart valve and pAF ablation surgery underwent monopolar RF ablation (Group A: n=20; Group B: n=75); with the beginning of a bipolar RF approach (see below) since March 2003 the monopolar ablation concept was applied during mitral valve (MV) surgery only (when the left atrium has to be opened anyhow). To create endocardial RF ablation lesions either the Thermaline® device (2001) or the Cobra® device (2002-2004) were used (both Boston Scientific Corporation, San Jose, USA). Monopolar RF ablation was performed with 100W RF power for two minutes (local temperature: 70°C). The first lesion completed the isolation of the right pulmonary veins (RPVs) from the inferior to the superior RPV using the left atriotomy. Isolation of the left pulmonary veins (LPVs) was performed with a semicircular ablation line close to the inferior, and another one around the superior LPV. These were connected by a transverse lesion across the posterior LA wall. The left atrial appendage (LAA) was sutured from the endocardial side in cases with LA enlargement (LA-diameter =56mm). Arrangements to avoid thermic esophageal injury were: (1) the ablation procedure was performed under direct view during conventional open heart valve surgery only, (2) the transesophageal echocardiogram (TEE) probe was removed during ablation, (3) local temperature was set at only 70°C (4) a dry compress was passed behind the LA before energy delivery, (5) the flexible ablation probe was adapted to the tissue without pressure and (6) cachectic patients were excluded.

*The bipolar RF ablation procedure* has been described previously [3,4]; a similar surgical concept using microbipolar RF coagulation to replace incisions of maze surgery has been reported before by Patwardhan et al [5]. Since March 2003 bipolar RF ablation surgery was performed using the bipolar Atricure® device (Atricure Inc., Cincinnati, USA) combined with CABG and since October 2003 concomitant to aortic valve replacement (Group A, B: n=19 vs. n=12). After start of cardiopulmonary bypass (CPB) isolation of the RPVs and the LPVs was performed by impacting the atrial tissue between the jaws of the Atricure® hand piece and then energy was delivered (local temperature 40-55°C). The procedure was terminated when the ablation and sensing unit (ASU) indicated that the tissue conductance was at least 10 sec below 2.5 Millisiemens. Then a purse-string suture was set at the posterior LA wall. The distal jaw was inserted through an incision in direction of the LPVs and ablation was performed after clamp-closure. Finally the distal jaw was inserted in direction of the RPVs and the connection line was completed. The LAA was sutured from the epicardial side in cases with LA enlargement (see previous)

Transthoracic echocardiogram (TTE) and standard 12-lead electrocardiogram (ECG) were routinely performed on admission and before discharge. TTE was performed by an experienced cardiologist. The preoperative LA size was determined from parasternal M-mode acquisitions at end systole using a Vingmed Vivid 5 (General Electric-Vingmed) system. Amiodarone administration was started with an intravenous 300 mg bolus before CPB termination followed by an infusion of 900mg/day for 3 days; after that, oral administration of 5x200 mg up to 7-10 g was begun (dependent of body weight), 1x200 mg/day followed for 3 months. In cases of amiodarone contraindication (e.g. amiodarone imcompatibility, thyroid disease), sotalol was given alternatively (first intravenous bolus of 10 mg, then 1 mg/kg for 24 hours; oral administration of 2-3x40-80 mg dependent of body weight for 3 months). An indication for amiodarone/sotalol termination was persisting bradycardia for =10 days. An indication for pacemaker implantation was persisting bradycardia for =2 weeks.  Early recurrence of AF was DC cardioverted after antiarrhythmic drug saturation and after exclusion of intracardiac thrombosis by TEE. During initial therapy and particularly in case of AF recurrence or bradycardia, patients were observed with continuous monitoring on the intensive or intermediate care unit (ICU). Heparin was given after resolution of postoperative bleeding. Patients with CABG, MV repair or bioprosthesis got cumarine for 3 months, patients with mechanical valves lifelong anticoagulation. All patients were restudied  3,  6,  9,  12, 18,  24 and 36 months  after  surgery  by  standard  12-lead  ECG  and  clinical examination. To confirm stability of SR one 24-hour-ECG registry was performed within the first 6 months.

### Statistical analysis

Quantitative data were normally distributed and described by arithmetic mean ± SD, qualitative distributed data were presented as absolute frequencies; for pAF and SR the relative frequency among all patients and some subgroups were calculated. Comparison of proportions was performed using exact Fisher Chi-Square-Test. Survival time was analyzed by the method of Kaplan-Meier, comparison of cumulative survival was performed using Log Rank Test. P-values <0.05 were considered to be statistically significant; analysis was performed with SPSS for Windows 11.5.1.

## Results

All relevant operative and postoperative data are outlined in  Table 3-5. All 126 patients left the OR either in regular SR or externally paced in DDD-mode. In general, Group B patients had a slightly faster recovery than Group A patients. Thirty-seven percent of Group A patients kept SR during hospital stay (Group B: 50%), 63% had early AF recurrence (Group B: 50%). DC cardioversion was performed with success in 16 of 32 Group A patients (Group B: 9 of 23 patients), 2 patients of Group A converted to SR spontaneously. At discharge 63% of patients of Group A (n=43) had SR (Group B: 66 %; n=37) (Table 5). No case of left atrial flutter was observed. Early mortality (<30 days) was 2.9% in Group A (n=2: one severe sepsis, one sudden cardiac death) and 0% in Group B. Autopsy was performed in both cases to exclude ablation related complications. Other complications were 9 cases of transient pulmonary malfunction (acute pneumonia: Group A n=3, Group B n=2; acute obstructive ventilation disorder: Group A n=3, Group B n=1), 2 cases of enteropathy (one each group), 2 transient strokes and 2 slight wound infections (Group A).

**Figure 1:**
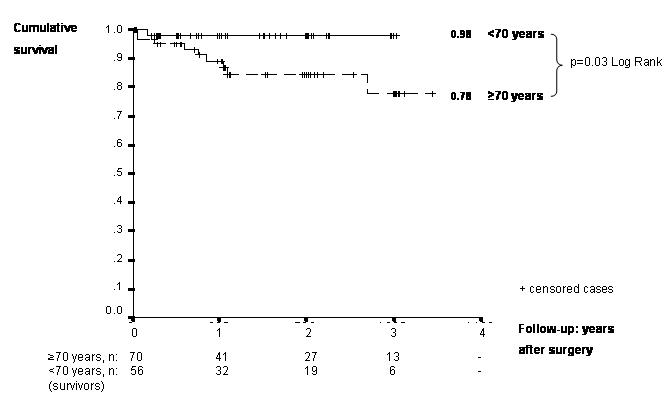
Kaplan-Meier cumulative survival curves of 126 patients, demonstrating a 78% cumulative survival after combined open heart and pAF ablation surgery of older aged patients (n=70) and a 98% survival in patients <70 years (n=56).

All patients were regularly restudied with follow-up of 18.5±12.0 months in Group A (Group B: 15.2±11.0 months). Administration of amiodarone/sotalol was stopped after 3 months in all patients. Late mortality occurred in eight cases (Group A, B: n=7 vs. n=1), which was related to 2 cardiac and 6 non-cardiac deaths. Cumulative survival at long-term follow up was 0.78 vs. 0.98 (p=0.03; Figure 1), NYHA-class improved from 3.0±0.5 to 1.4±0.5 (Group A) and from 3.0±0.5 to 1.5±0.5 (Group B; p<0.01). In general at 6 months follow-up patients with stable SR (71 of 94) had a significantly higher NYHA-class improvement (78% NYHA 1, 22% NYHA 2), than patients with persisting pAF (13% NYHA 1, 87% NYHA 2; p<0.001). At 12-months follow-up 73% patients of Group A patients presented stable SR (Group B 78%; Table 4).

## Discussion

Besides other variables cardiac disease and particularly age highly predisposes atrial musculature to develop and maintain atrial fibrillation [1]; thus mid- and long-term results of surgical AF treatment may also depend on age and cardiac disease. The presented study data describe our experience with RF ablation surgery in a selective group of older aged patients and the effects on survival, functional class and SR conversion rate. In the current literature reliable data on this subject are scarce. However, as in Germany and other countries the number of open heart procedures in older aged patients is increasing dramatically, this topic is of progressive importance; in 2003 notably 40% of all patients hospitalized for open heart surgery in Germany were 70 years or older, with a 5-fold increase of procedures in the 7th decade and a 14-fold increase in patients older than 80 years, compared to 1990 [6]. It must be anticipated, that this trend is influencing directly the number of older aged patients scheduled for open heart surgery with concomitant atrial fibrillation, because AF is predominantly found at advanced age, affecting approximately 5-10% of people in the 7th decade and 10-15% of people older than 80 years [1]. Particularly permanent AF, which is normally the final mode in which non-permanent AF cumulates[7], deteriorates the prognosis of old people: it doubles the mortality rate and increases dramatically the morbidity, as (1) a loss of rapid ventricular rate without adequate “AV-synchronization” and atrial contraction results in reduced cardiac output and potential heart failure, (2) the risk of stroke by blood pooling in the atria is increased, and (3) stroke prevention forces systemic anticoagulation with the risk of bleeding [1]. For that reasons concomitant treatment of pAF during open heart surgery in aged patients has become a great challenge and reliable concepts are required, which can be applied during open heart surgery particularly in the older aged with low risk, satisfactory success-rates regarding SR conversion rate and secondary survival.

Compared with the literature our data confirm the experience that longstanding pAF is predominantly a disease of the elderly, particularly of older aged women. In the literature a gender difference has been described, but the etiology of this finding is still unclear [1]. Normally men develop AF at 1.5 times the rate of women. However, possibly because of the greater longevity in women, the minority of AF cases is found in men. It is estimated that 53% of all people affected by AF are female [8]. Among patients scheduled for open heart surgery, men are more often hospitalized with isolated coronary artery disease, which is not frequently combined with pAF [2,4]; in contrast a high ratio of women is hospitalized with severe heart valve disease, in which a higher occurrence of pAF has been described [2,4].

We interpret the clinical results of ablation surgery particularly on the basis of researches of others: AF wavelets sustained by foci located inside the PVs were blocked by the created lesions [9-13]. The antiarrhythmic drug protection using amiodarone or sotalol supported SR during the unstable initial stage, which was approximately 3 months; amiodarone was given as first choice antiarrhythmic drug to reduce postoperative recurrence of AF according to the data of Roy at al [14] and the clinical experience of others [15]. However, AF recurred soon after surgery in more than half of the patients because the refractory period of the atrium was still shortened.

Our data further indicate that the described concepts (including a monopolar endocardial RF ablation technique during mitral valve surgery and a bipolar approach in non-mitral valve cases) can be performed in a selective group of older aged patients with low risk and satisfactory success-rates regarding survival, improvement of NYHA class and conversion rate to SR after surgery. Even if young patients had a slightly faster recovery than older aged patients, the postoperative SR conversion rate was of no significant difference; perioperative complications were low and postoperative management including antiarrhythmic drug administration and early DC cardioversion under anticoagulation were tolerated well. We therefore suppose that the described strategies are effective and can be recommended as reliable treatment strategies to cure pAF during open heart surgery in patients with advanced age.

## Limitations

This study consists of a heterogeneous patients group regarding the type of pathology and the type of RF ablation technique, what limits the evidence. The data were not evaluated under randomized conditions, further the groups were not matched. For rhythm evaluation no 7-days Holter-monitoring and only one 24-hour-ECG registry was performed within 6 months, at long-term follow-up (6-36 months) only 12-lead ECG was used. Patients with severe reduced left ventricular function were excluded, which explains the good LVEF in both groups despite structural heart disease and pAF.

## Figures and Tables

**Table 1:**
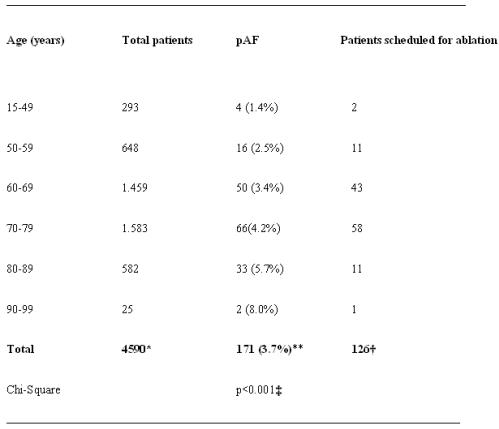
Prevalence of pAF (≥6months) among 4,500 open heart cases

*ratio male/female: 67.6% (n=3103) vs. 32.4% (n=1487); pAF among men and women: 88 of 3103 (2.8%) vs. 83 of 1487 (5.6%), p<0.001; **male: n=88, female: n=83; †male: n=65, female: n=61; ‡pAF in older aged (70-99 years) vs. young (<70 years) patients: 101 of 2190 [4.6%] vs. 70 of 2400 [2.9%].

**Table 2:**
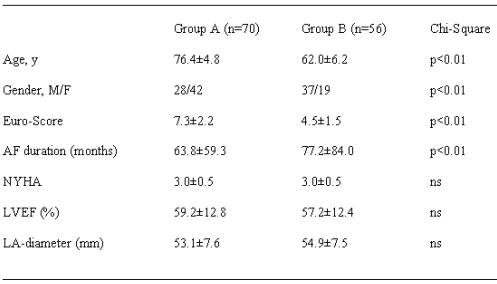
Patient characteristics (n=126)

LVEF, left ventricular ejection fraction; NYHA, New York Heart Association

**Table 3:**
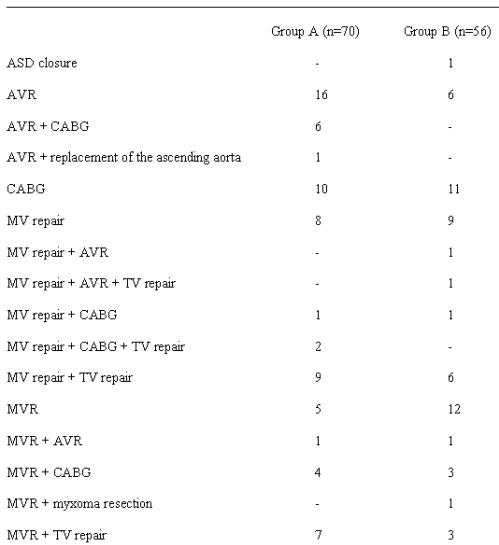
Surgical Procedures (n=126)

ASD, atrial septal defect; AVR, aortic valve replacement; CABG, coronary artery bypass grafting; MVR, mitral valve replacement; TV, tricuspid valve

**Table 4:**
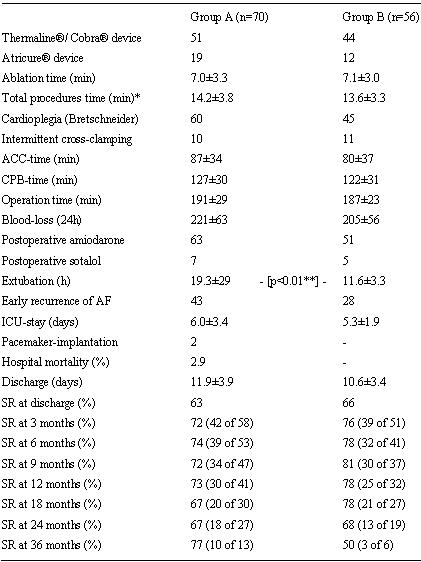
Surgical and early postoperative data (n=126)

ACC, aortic cross clamping; CPB, cardiopulmonary bypass; ICU, intensive/intermediate care unit; *including adjustment of the equipment and a precise adaption of the probe to the tissue (Thermaline® /Cobra®) and preparation of the PVs (Atricure®); ** exact Fisher Chi-Square-Test

**Table 5:**
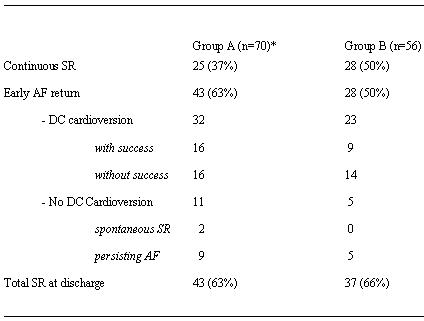
Early postoperative data (time of operation until discharge; n=126)

*early deaths n=2
